# Editorial to the Special Issue on “Polyamine Metabolism in Health and Disease: Potential for Polyamine-Targeted Therapies and Prevention”

**DOI:** 10.3390/medsci11030053

**Published:** 2023-08-19

**Authors:** Tracy Murray Stewart

**Affiliations:** Sidney Kimmel Comprehensive Cancer Center, Johns Hopkins University, Baltimore, MD 21287, USA; tmurray2@jhmi.edu

To introduce this Special Issue, I refer the reader to the timely review by Zahedi and colleagues [1], which highlights the various roles of polyamines and their metabolism in the maintenance of normal physiological homeostasis as well as the consequences resulting from metabolic abnormalities and altered tissue polyamines levels. The causes of metabolic perturbations, including genetic variants or mutations, tissue injury, toxins, and pathogens, are discussed along with their roles in supporting both health and disease. This review includes a valuable compendium of the regulatory mechanisms of several key polyamine metabolic enzymes and describes current knowledge on the ways in which polyamines interact with other molecules in the cell, including nucleic acids, translation factors, free radicals, and certain proteins. The “double-edged” sword is an important central theme of this article, which accurately depicts the diversity of outcomes associated with context-specific polyamine pathway alterations.

Of these, the dysregulation of polyamine metabolism due to inherited genetic variants in the pathway has, until recently, been unappreciated and understudied. For this Special Issue, Michael and colleagues [2] compiled a case report describing the two most recent patients identified as having Bachmann–Bupp syndrome, the second of these “polyaminopathies” to be characterized. This report additionally introduces the reader to the International Center for Polyamine Disorders (ICPD), which has been established to facilitate research opportunities as well as provide education and medical care to affected families. Thus far, a total of five syndromes have been identified as resulting from pathogenic gene variants in different parts of the polyamine pathway, and the authors speculate that at least three times as many more remain to be discovered. The ICPD also serves as a repository of samples and cell lines from polyaminopathy patients and their family members, providing a valuable resource for the study of these rare disorders. 

Further emphasizing the importance of polyamines in enabling cellular proliferation, an investigation into the interaction of polyamines with the mTOR pathway in breast cancer is reported in a research communication by Akinyele and Wallace [3]. Using genetic and pharmacological inhibition of the mTOR pathway along with polyamine biosynthesis inhibition, the authors suggest that cancer cell proliferation is supported by crosstalk between the pathways and propose combined inhibition of the two pathways as a treatment strategy. Evidence is presented indicating reduced translational initiation in the antiproliferative mechanism.

The regulation of polyamine homeostasis is critical to all forms of life. In a comprehensive review by Krysenko and Wohlleben [4], nitrogen assimilation and its control in various species of bacteria are discussed. This includes the use of polyamines, as well as the monoamine ethanolamine, as a source of essential nitrogen under conditions in which its abundance is otherwise limited. However, as with all cell types, high levels of environmental polyamines can exert toxicity, with variability among bacterial species. Therefore, the ability to metabolize polyamines also serves to control toxicity from high intracellular levels. The known polyamine metabolic pathways at play in both commensal and pathogenic bacteria are described, as are the currently identified polyamine transport mechanisms. Also summarized is the abundance of individual polyamines in certain habitats as well as the presence/absence of certain polyamines and their intracellular concentration ranges in multiple microbial species, making this article a valuable resource. 

In contrast, the intricacies of polyamine transport into and within cells of higher organisms are still being elucidated. Stump and colleagues [5] discover a putative role for Chmp1, a vesicular trafficking protein, in polyamine transport. Using a *Drosophila melanogaster* assay system, it is shown that overexpression of Chmp1 could protect against the cytotoxic drug Ant44, which is known to use the polyamine transport system for internalization. Their results suggest a negative regulatory role for Chmp1 in polyamine transport that may include specificity for certain polyamines. As the CHMP1A protein is also overexpressed in human cancer cells with disrupted polyamine transport, this study adds support to the current models proposing that imported polyamines are sequestered into vesicles.

Along these same lines, Holbert and colleagues [6] compare the transport of spermine analogue-based nanoparticles with the parent molecules in human lung cancer cell lines. The nanoparticle strategy is based on the assumption that the polyamine transport system (used by the parent analogue) is independent of general endocytosis (used by nanoparticles). However, the authors discover substantial overlap between these mechanisms, supporting a model of polyamine import that includes endocytosis. 

Regarding therapeutic strategies targeting polyamines, difluoromethylornithine (DFMO) is likely the most well studied pharmacological modulator of the polyamine pathway. DFMO acts as a suicide inhibitor of the first rate-limiting enzyme in polyamine biosynthesis, ornithine decarboxylase (ODC), thereby limiting intracellular polyamine concentrations. In a research article by El Naggar and colleagues [7], DFMO is demonstrated to improve the response of ovarian cancer cells to the PARP inhibitor rucaparib by increasing chemotherapy-mediated DNA damage. Considering the well-established clinical safety profile of DFMO, the inclusion of polyamine-blocking therapy as an adjunct to chemotherapy in ovarian cancer should be considered. 

The catabolic oxidation of spermine is induced in several pathological states and is believed to contribute to the carcinogenic process by generating oxidative stress. Inhibition of spermine oxidase (SMOX) has therefore become a strategy for the chemoprevention of cancer in high-risk populations and may also be beneficial in protecting against SMOX-mediated tissue injury in other pathologies. In this issue, Furbish and colleagues [8] describe the discovery and characterization of novel inhibitors with enhanced specificity for SMOX over structurally related amine oxidases. The specificity and potency of these molecules for SMOX inhibition are significantly improved compared to the commonly used polyamine oxidase inhibitors and will be instrumental for the further development of this line of research.

Rounding out this Special Issue is an excellent review on polyamine depletion and tumor immune microenvironment remodeling, provided by Chin and colleagues [9]. While polyamine depletion strategies directly affect the ability of tumor cells to proliferate, it is becoming clear that they also impact components of the tumor microenvironment, particularly immune cells, resulting in a reduction in immunosuppression and enhanced therapeutic response. This review summarizes the current understanding of this response in the various immune cell types and discusses ways in which polyamine blocking strategies have been combined with other treatment modalities to improve efficacy and survival.

## Data Availability

Not applicable.
